# Assessing the involvement of long-term memory in working memory

**DOI:** 10.3758/s13423-025-02774-7

**Published:** 2026-01-20

**Authors:** Julie Pougeon, Clément Belletier, Pierre Barrouillet, Valérie Camos

**Affiliations:** 1 Département de Psychologie, Université de Fribourg, Rue P.-A. de Faucigny 2, 1700 Fribourg, Switzerland; 2 Laboratoire de Psychologie Sociale et Cognitive, Université Clermont-Auvergne, 17 rue Paul Collomp, 63037 Clermont-Ferrand, France; 3 Université de Genève, Geneva, Switzerland

**Keywords:** Working memory, Long-term memory, Episodic memory, Attention, Residual

## Abstract

Complex span tasks are working memory (WM) tasks in which participants maintain series of items (e.g., letters) for further serial recall while performing a concurrent task (e.g., parity judgement on digits). It has been shown that even pushing the demand of this concurrent task at its individual limits strongly reduces, but does not totally abolish, memory performance. A small memory residual of about one item remains. The present study aimed at testing the hypothesis that this residual is retrieved from long-term memory (LTM). For this purpose, two experiments compared the size of memory residual either through immediate recall or after a 1-min delay filled with a backward counting task. If it is retrieved from LTM, a substantial part of this residual should still be accessible after the delay. Although this delay reduced the immediate memory residual, about two-thirds of this residual was still retrievable when the complex span task was performed under concurrent articulation. These findings confirmed that when processing almost entirely captures attention, memory residual mainly relies on LTM. However, the fact that forgetting rate during the complex span task was far larger than during the subsequent delay weakens WM theories suggesting that memory items are offloaded in activated LTM when attention is switched away. We suggest that our findings are more compatible with the short-term transient storage hypothesized by the synaptic theory of WM.

## Introduction

Working memory (WM) has usually been defined as a cognitive system devoted to the temporary maintenance of information in view of its processing in on-going cognition (Baddeley, 2007). The most significant characteristic of this system is probably its small capacity of storage. Limited to approximately seven items when no processing is to be carried out simultaneously (Miller, 1956), this memory capacity rapidly decreases as the cognitive demand of concurrent processing increases (see Barrouillet & Camos, 2015, 2021, for reviews), a phenomenon that led many theoreticians to assume some resource-sharing between storage and processing functions in WM (e.g., Baddeley et al., 2021; Barrouillet & Camos, 2015, 2021; Case et al., 1982; Cowan et al., 2021).

In a series of recent studies, Barrouillet et al. (2024; see also Belletier et al., 2021; Pougeon et al., 2024) explored the limits that this resource sharing imposes on the human cognitive system when individuals are performing at their maximum capacity, either for processing or for storage. The goals of these studies were to assess humans’ ability to maintain some memoranda in mind while performing at their best in a concurrent task, and conversely, their processing capacity when maintaining a short-term memory load at their own span. To answer these questions, we used complex span tasks in which participants were presented with series of letters for immediate serial recall, the presentation of each letter being followed by a 6-s period of processing consisting of a parity judgement task on digits appearing successively on screen. We first assessed, through titration procedure, both memory and processing spans for each participant (i.e., the maximum number of letters they were able to recall in correct serial position and the maximum number of digits they were able to judge in each 6-s interval, respectively). Then, they performed both tasks set at span in isolation before performing them in combination in the complex span task, both tasks still being set at the individual’s span. The critical manipulation was to ask participants to prioritize either processing or storage. The compliance on this prioritization instruction was assessed through what is called the “perfect trial procedure”. Participants had to perform five trials in which their memory performance in the dual task was at least equal to their performance in the single task, and five trials in which the same requirement concerned processing performance. The measure of interest was the residual performance in the non-prioritized task, that is the number of letters recalled in correct serial position or the number of digits participants were able to correctly process in the 6-s intervals.

It appeared that the human cognitive system privileges processing over storage, processing residuals being far larger than memory residuals (Barrouillet et al., 2024). However, even in the extreme condition of a processing demand pushed at its maximum, maintenance capacities are not totally abolished. There is still a memory residual of about one letter. Due to the controlled compliance on the prioritization instruction in the perfect trial procedure, Barrouillet et al. (2024) surmised that processing prevented any active maintenance of the memoranda in WM, and consequently hypothesized that this residual came from long-term memory (LTM). The authors noted that such a hypothesis is in line with Cowan et al. (2012) who tested several computational models to fit experimental data in verbal WM studies involving words. They observed that a model successful in accounting to recall performance from WM needed the addition of an LTM component of one word.

The aim of the present study, beyond replicating Barrouillet et al.’s (2024) results, was to test the hypothesis of the LTM origin of the memory residual, thus potentially providing empirical evidence to Cowan et al.’s (2012) proposal. We used for this purpose the same general procedure as in Barrouillet et al. (2024) involving serial recall of letters and parity judgement tasks. After titration, and single tasks, participants performed a dual task with, due to our exclusive interest in memory residual, prioritization on processing. These tasks were performed without (Exp. 1) and with concurrent articulation (Exp. 2). However, memory residual was assessed either through immediate recall after the last processing episode, as in Barrouillet et al., or delayed recall, a 60-s backward counting task intervening between the end of the last processing episode and recall. A stringent criterion for the hypothesis of a memory residual retrieved from LTM would be an unchanged performance between immediate and delayed recall. By contrast, if the memory residual observed when prioritizing processing is retrieved from WM and not LTM, this residual should vanish during the 60-s backward counting task and fall down to zero.

## Experiment 1

### Method

#### Participants

Twenty undergraduate students from the University of Fribourg (Switzerland) (17 women, three men) ages 8 to 23 years (*M* = 21.00 years, *SD* = 0.92), with normal or corrected-to-normal vision, and no dyslexia or dyscalculia, received course credits or gift voucher for their participation. The internal institutional review board approved the ethics of this study (application ID: 2022-792).

The experiments were preregistered, including participants recruitment procedure. We planned on recruiting a minimum of 20 participants^1^, and to follow the sequential Bayes factors design with maximal *n* procedure (Schönbrodt & Wagenmakers, 2018) in which a minimal number of participants is first recruited, and BFs are computed according to planned analyses. If the BFs do not reach an a priori–defined level of evidence, participants are sequentially added and BFs are analyzed at each step, until the BFs threshold is met, or the defined maximal *n* of participants is reached. We aimed at obtaining a BF_inclusion_ or BF_exclusion_ superior to 3 for the main effect of recall type (immediate vs. delayed recall test). We had planned to add four participants if the threshold was not reached for each step, up to a limit of 40 participants. Overall, we had to recruit 25 participants because five decided to stop before the end of the experiment. As planned, we performed a nonparametric *t* test Bayesian analysis on the 20 participants who completed the task. The BF value was superior to the preestablished threshold and we therefore conducted our analyses on these participants.

#### Material

The tasks were administered on a computer using PsychoPy software (Peirce, 2007). The items to memorize were all the consonants, except “W”, which is trisyllabic in French. The digits from 1 to 9 were used during the parity judgement task. Instructions for each task were presented on screen. Participants completed and signed a consent form before starting the experiment. Four experimenters were randomly assigned to the same number of participants. During the experiment, they remained seated in the experimental cubicle, in such a way that they could not see the participant’s computer screen but remained available to answer any questions regarding instructions.

#### Procedure

First, individual memory spans (the maximum number of letters participants were able to recall) and processing spans (the maximal number of digits participants managed to judge during a number of 6-s intervals equal to their memory span) were assessed through titration procedure. These titrations were followed by memory and processing single tasks set at span. Finally, participants completed the dual task with both memory and processing components set at span while giving priority to processing, with recall being either immediate or delayed for 60 s by a backward counting task, a traditionally used task for delayed recall test.

#### Titration

The memory titration used a staircase procedure involving eight steps with two trials per step, beginning with four letters in the first step. If the participant managed to recall at least 90% of the letters in correct serial position in the average of the two trials of a given step, the number of letters was increased of one unit in the next step, or otherwise decreased of one unit. The procedure ended at Step 8, unless the number of items was the maximum obtained by the participant, in which case the procedure continued until the participant failed.

**Fig. 1:**
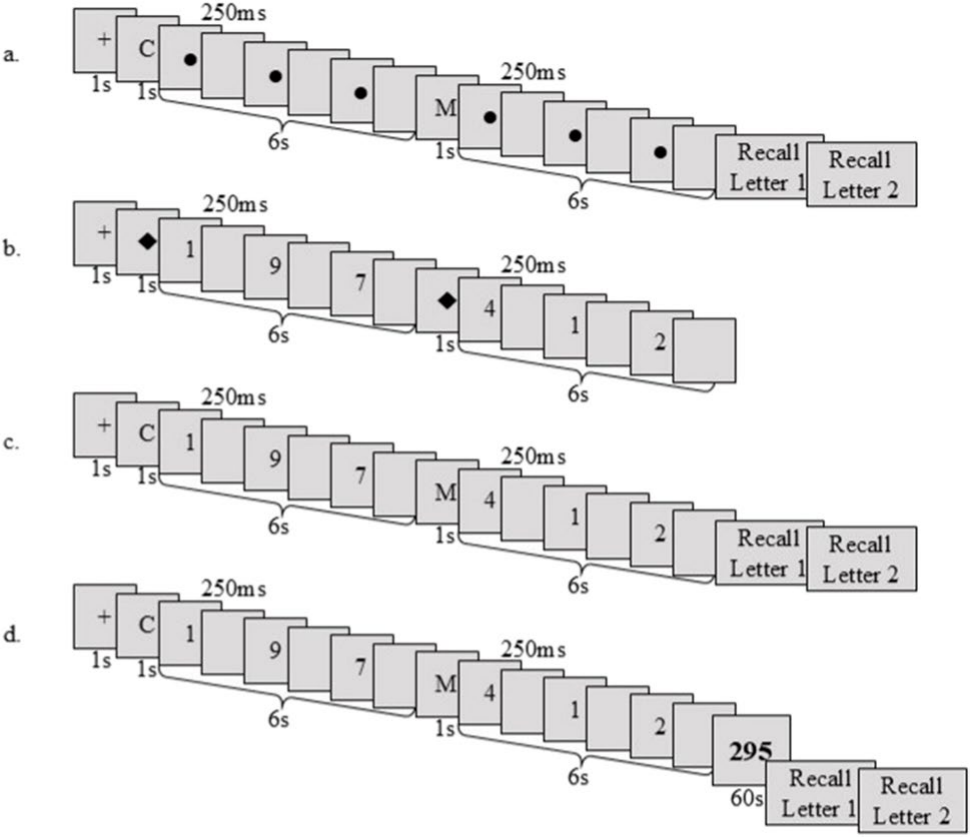
Illustration of the memory and processing tasks in single task and dual task with immediate recall and delayed recall conditions. Time course of the successive events appearing on screen for the memory titration and memory single task **(a)**, the processing titration and processing single task **(b)**, and the dual task with immediate **(c)** and delayed recall **(d)** in which participants performed a 60-s backward-counting task from a three-digit number displayed on-screen

A cross appearing on screen accompanied by a beep sound indicated the beginning of a trial. Then, each letter appeared on screen for 1 second and was followed by a 6-second interval during which a circle was blinking (Figure 1). This circle was a placeholder replacing the digits that would appear during the dual task. After the last 6-second interval, a second beep indicated the end of the presentation of the letters, and “Recall Letter 1” appeared on screen. The letter typed by the participant quickly appeared on screen and was replaced by a “Recall Letter 2” screen, and so on until all the letters have been recalled. Participants were instructed to enter the letter “O” if they had forgotten a letter. Memory span corresponded to the maximum number of letters for which the criterion of 90% correct recall was reached.

This memory titration was followed by a processing titration in which participants had to judge the parity of digits appearing successively on screen in a number of 6-s intervals equal to their memory span. The same staircase procedure was used as well as the same accuracy criterion, beginning with four digits per 6-s interval. If an average of at least 90% of the digits was correctly judged in the two trials of a given step, the number of digits per 6-s interval was increased of one unit in the next step, and otherwise decreased of one unit. Titration stopped at the eighth step, unless the number of digits correctly judged corresponded to the maximum level obtained by the participant, in which case the procedure continued until the participant failed.

In each trial, a cross appearing on-screen with a beep sound indicating the beginning of the task. Then, diamonds (placeholders for the letters in the forthcoming dual task) appeared for 1 s, each diamond being followed by a 6-s interval during which participants had to judge the parity of digits (Fig. 1) by pressing the right or the left arrow key for “even” and “odd” responses, respectively. Each digit was followed by a fixed interval of 250 ms, during which participants could still enter their response. The display time (in ms) of each digit was determined by the following formula: (6,000 − (250 × *n*))/*n,* in which *n* is the number of digits to be presented per 6-s interval. After the last 6-s interval, a beep announced the end of the trial. Processing span corresponded to the maximum number of digits per 6-s interval for which the 90% correct criterion was reached.

Both memory and processing titrations were preceded by three training trials, each trial having three letters to memorize for the memory training task and three sequences of three digits to judge for the parity judgement task.

#### Single task

After titrations, participants performed five trials of a single memory task and five trials of a single processing task, with a number of items to memorize or process set at span.

#### Dual task with immediate and delayed recall conditions

Finally, participants performed the dual task that combined the single memory and processing tasks. Both the number of letters to memorize and of digits to process were set at span. After a ready signal (a cross) appearing on screen from 1 s, each letter appeared for 1 s and was followed by a 6-s interval for parity judgement. After the last interval, the recall phase was either immediate or delayed for 60 s by a backward counting task in which participants were asked to count backwards and aloud by threes from a three-digit number randomly chosen between 200 and 500 and displayed on-screen with the instruction to count. The end of the counting task was indicated by a beep and followed by the same recall phase as described above.

During this dual task, participants were asked to prioritise the processing component of the task by achieving 10 “perfect” trials in 40 attempts. We deemed a trial as perfect when processing performance was equal or better than that obtained in the single processing task. The type of recall (either immediate or delayed) for each trial was distributed in a pseudorandom way^2^. Our criterion was to obtain five perfect trials with an immediate recall and five perfect trials with a delayed recall for each participant. Participants were continuously informed how many perfect trials they had completed out of the 10 required, without mentioning the number of successful trials per recall condition. They were also told the number of remaining attempts out of 40. The experiment continued until participants had achieved at least five perfect trials in both types of recall or until 40 attempts had been achieved. This dual task was preceded by three training trials with an immediate recall and three trials with a delayed recall. Each trial consisted of three letters to memorise and three digits to judge for 6 s after the presentation of each letter.

Memory residuals were expressed, for immediate and delayed recall conditions, in terms of percentage of correct answer (i.e., the ratio between the number of letters recalled in correct serial position and the number of letters presented, that is, the span) as well as in terms of number of letters by multiplying, for each participant, their percentage of correct answers in the residual by their memory span.

### Results and discussion

All participants achieved the five perfect trials required in each recall condition (immediate and delayed) of the dual-task and were therefore included in the following analyses. On titration, participants had an average memory span of 6.85 letters (95% CI [6.20, 7.45]) and an average processing span of 6.60 digits judged in 6 seconds (95% CI [6.09, 7.11]). All analyses were performed with JASP Software (Version 0.19.1; JASP Team, 2024), and data are available on OSF.

We analysed the mean percentage of correct responses across trials for each participant in processing and memory performance in single tasks and in the perfect trials for the dual task. These percentages were corrected for guessing using the formula proposed by Diamond and Evans (1973): “*pcorr = praw – (perrors/(k − 1))”* in which *pcorr* is the percentage corrected for guessing, *praw* the percentage of correct responses, and *perrors* the percentage of incorrect responses excluding omissions; *k* is the number of possible answers, which was 2 for the processing task (even or odd) and 19 for the memory task (all the consonants excluding *w*). For the dual task, analyses were carried out on the first five perfect trials achieved in each recall condition for each participant.

Concerning the parity judgement task, the mean percentage of correct responses was 86.3% (95% CI [84.0, 88.7]) in the single task, 93.6% (95% CI [91.7, 95.5]) in the dual task with immediate recall, and 94.2% (95% CI [92.3, 96.1]) with delayed recall. A Bayesian paired-sample *t* test^3^ provided anecdotal evidence that there was no difference in processing accuracy between immediate and delayed recall conditions, BF_10_ = 0.35.

**Fig. 2:**
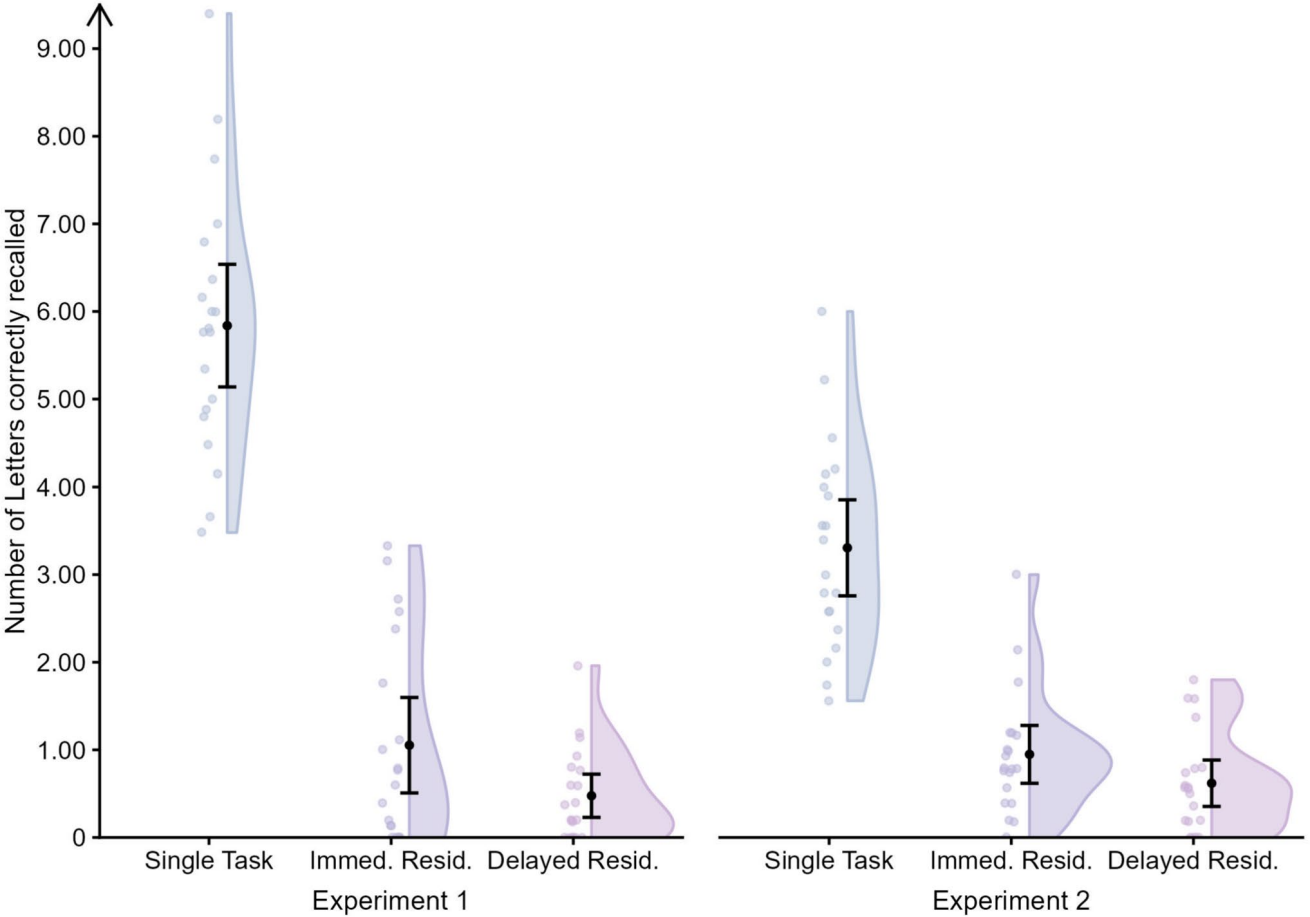
Number of letters correctly recalled in correct position in the single task and the perfect trials of the dual task (residuals) in the Immediate (Immed. Resid.) and Delayed (Delayed Resid.) recall conditions for Experiment 1 (without concurrent articulation) and Experiment 2 (with concurrent articulation)

Concerning the memory task, non-parametric Bayesian paired-sample t-tests using Bayesian Wilcoxon signed-rank tests (with five chains of 5,000 iterations)^4^ showed that the percentage of letters recalled in correct serial position was higher in the single than in the dual task with immediate, BF_10_ = 6.15×10^3^, and delayed recall, BF_10_ = 6.82×10^3^. Moreover, recall performance was lower in the delayed than immediate recall condition, BF_10_ = 8.19 (Table 1). Nonetheless, there was strong evidence that the mean memory residual in terms of number of letters recalled after the 60-s delay (0.48 letters) was higher than zero, BF_10_ = 1040.24, as was the memory residual of 1.05 letters observed with immediate recall, BF_10_ = 924.09 (Fig. 2).

**Table 1:** Mean (and *SD*) percentage and number of letters recalled in correct serial position in single task and dual task for the immediate and delayed recall conditions

Concurrent articulation	Recall condition	Percentage of recall	Number of letters recalled
Without (Exp. 1)	Single	85.5 (14.4)	5.84 (1.50)
	Immediate	16.5 (20.5)	1.05 (1.16)
	Delayed	6.9 (7.2)	0.48 (0.53)
With (Exp. 2)	Single	83.0 (14.1)	3.31 (1.17)
	Immediate	26.3 (22.6)	0.95 (0.71)
	Delayed	17.4 (16.7)	0.62 (0.57)

Finally, even without taking serial position into account (i.e., free recall scoring), no evidence was gathered for or against a difference between the immediate (23.4%; *SD* = 26) and the delayed (12.4%; *SD* = 10) recall performance, BF_10_ = 1.84. These percentages corresponded to memory residuals of 1.51 (*SD* = 1.48) and 0.86 (*SD* = 0.78) letters, respectively, which were both higher than 0, BF_10_ = 3.47×10^3^ and 2.90×10^3^, respectively.

Before discussing the implications of these findings, a second experiment reassessed residuals in immediate and delayed recall, while participants concurrently performed a concurrent articulation. Several WM models describe a specific maintenance mechanism for verbal WM that coexists with the attentional system (Baddeley et al., 2021; Barrouillet & Camos, 2021). Blocking this mechanism with a concurrent articulation should allow a more precise estimation of memory residual after attentional capture by a demanding processing task.

## Experiment 2

### Method

#### Participants

Participants were 20 undergraduate students (19 women, one man) from University of Fribourg (Switzerland). They were between 18 and 26 years of age (*M* = 21.30 years, *SD* = 1.69), with normal or corrected-to-normal vision, and no dyslexia or dyscalculia.

The number of participants was determined by a procedure like the one we used in Experiment 1. Ultimately, we had to recruit 21 participants because one did not reach the preregistered criterion of at least four perfect trials achieved in the dual task.

#### Material and procedure

The method and materials were the same as in the first experiment, except that participants performed a concurrent articulation during all the tasks. They started to say aloud “*ba-bi-bou*” at the beginning of each trial indicated by the beep and ended up when the recall of letters or the backward counting task started, indicated by the second beep.

### Results

The titration revealed a mean memory span of 4.00 letters (95% CI [3.39, 4.61]) and a mean processing span of 5.75 digits (95% CI [5.11, 6.39]) judged in 6 s. As expected, the memory span was lower than in the first experiment in which participants did not perform a concurrent articulation, BF_10_ = 1.6×10^5^. Statistical analyses were like those conducted in the first experiment. All participants achieved the required perfect trials.

Concerning the processing task, the mean percentage of correct responses corrected for guessing was 84.3% (95% CI [81.2, 87.4]), 93.6% (95% CI [91.0, 96.3]) and 94.6% (95% CI [92.1, 97.0]) in the single task, and the dual task with immediate and delayed recall conditions, respectively. A nonparametric Bayesian paired-samples *t* test^5^ provided anecdotal evidence for an absence of difference between immediate and delayed recall conditions, BF_01_ = 2.08.

Concerning memory performance in terms of percentage of letters recalled in correct serial position, Bayesian nonparametric paired-sample *t* tests^6^ provided strong evidence for poorer recall in the dual task with immediate and delayed recall than in the single task, BF_10_ = 2.89×10^3^ and BF_10_ = 1.25×10^4^, respectively. Most importantly, recall performance was higher in the immediate than the delayed recall condition, BF_10_ = 3.97 (Table 1). As in Experiment 1, an additional nonparametric Bayesian one-sample *t* test indicated that the memory residual in terms of letters recalled in correct position was greater than 0, BF_10_ = 3.48×10^3^, and BF_10_ = 1.67×10^3^, for the immediate and delayed recall condition, respectively (Fig. 2).

As in Experiment 1, recall was rescored without taking serial position into account. Recall performance was higher in immediate (39.1%, *SD* = 22.9) than in delayed (26.0%, *SD* = 19.6) recall condition, BF_10_ = 64.50; the number of letters being higher than 0 in both immediate (1.42, *SD* =.76) and delayed (0.93, *SD* =.66) recall conditions, BF_10_ = 7.53×10^3^ and 9.75×10^3^, respectively.

## General discussion

The aim of the present experiments was to shed light on the origins of the small memory residual observed in WM complex span tasks when participants must concurrently perform a demanding task at their maximum capacity (Barrouillet et al., 2024; Belletier et al., 2021). Our hypothesis was that this residual is retrieved from LTM. The extreme version of this hypothesis would predict an unchanged memory residual between immediate recall, as traditionally used in WM studies and in Barrouillet et al.’s study, and a recall delayed by an extended 1-min-long intervening task.

First, the present study replicated the findings of Barrouillet et al. (2024) and Belletier et al. (2021), showing that a maximum level of attentional capture induced by a concurrent task performed at individual’s maximum capacity does not totally abolish storage capacities in WM. A memory residual remains but does not exceed a single letter. Second, our two experiments provided evidence that adding a 1-min delay reduced the size of this memory residual, probably because of a high level of interference introduced by the counting backward task. However, this residual was not abolished, as it could have been expected if it was entirely retrieved from WM. The memory residual after such a delay was of 0.48 and 0.62 letter when the task was performed without and with articulatory suppression, respectively. Such residuals were not negligeable (i.e., statistically higher than 0). They corresponded respectively to 46% and 65% of the residual observed when recall was immediate. These findings confirm the close dependence between processing and storage in WM (Barrouillet & Camos, 2021). When priority is given to processing, storage capacities fall to about a single retrievable item. Our results moreover indicate that about half of this residual is attributable to LTM. This means that 6-s episodes of an almost continuous occupation of attention by processing are enough to virtually eliminate all content in WM, only a small, but surprisingly resistant, residual being retrievable. These results have implications for current models of WM and our understanding of WM functioning.

Several models assume that WM is that part of LTM activated above threshold (Cowan, 1988; Cowan et al., 2021; Oberauer, 2002, 2009; Unsworth & Engle, 2007; for a review, see Logie et al., 2021). According to these models, among this activated LTM, a small number of items (about four) would be maintained in a privileged and highly accessible state in some focus of attention, zone of direct access, or primary memory. However, when attention is switched away from these items, they would fall in activated LTM, from which they could be retrieved. For example, Superbia-Guimarães and Cowan (2023) have recently assumed that there exists some transfer mechanism called “offloading” that would strategically displace items from the focus of attention to activated LTM in an easily retrievable form, with the benefit of freeing attention up for other tasks. Note that the authors mention that this offloading process would not be attention free, the focus of attention holding “pointers” to information held in activated LTM. This hypothesis of a short-term memory containing pointers to LTM addresses was already present in Nairne’s (2002) unitary view of memory, but has been recently renewed by neuroscientific investigations. Studies on selection and perception of visual objects have established the involvement of different brain areas on object identification on the one hand, and object individuation on the other, the former in the superior intraparietal sulcus (IPS) and higher visual areas, the latter in the inferior IPS (Xu & Chung, 2009). Object individuation seems to rely on load-dependent, but content-independent neural activity that remains unaffected by the complexity of the objects stored in WM and is only sensible to their number. Awh and Vogel (2025) have recently suggested that these load signals reflect the deployment of pointers binding item representations to the current surrounding context. According to Superbia-Guimarães and Cowan, it is this pointer-indexing system that would allow the retrieval of items offloaded in LTM.

There is no doubt that performing a parity judgement task at individual’s maximum capacity switches attention away from memory items, which should therefore be offloaded in LTM. However, even if it can be admitted that processing in this case is so demanding that even mere “pointers” cannot be maintained, it is difficult to imagine that memory traces offloaded in activated LTM would vanish in delays as short as 6 s. Should we not expect that an activated “long-term” memory is capable of preserving information for more than a few seconds? However, in Experiment 1, for example, participants were presented with an average of 6.85 letters, but only recalled 1.05 of them in immediate recall. It seems that the forgotten letters were not stored in such an activated LTM. This rate of forgetting (85% of the information lost in about 40 s) proved far larger than that occurring during the 1-min intervening counting task, after which about half (and even 65% in Exp. 2) of the memory residual observed in immediate recall was still accessible. If we assume that what can be retrieved from the residual after 1 min reflects LTM storage, the rate of forgetting during the complex span task suggests that letters have not been offloaded in activated LTM, otherwise they would have been more accessible.

An alternative hypothesis to the LTM storage of items from which attention has been switched away might be provided by the synaptic theory of WM (Mongillo et al., 2008). This theory posits that these items can be retained by short-term plasticity mechanisms that transiently modify in prefrontal cortex synaptic weights between the neurons that code for these items. Because these synaptic modifications disappear relatively slowly (about 1 s), memory traces could be reactivated. Accordingly, Rose et al. (2016) were able to produce a reemergence of the specific pattern of brain activity associated with a passively retained WM item, and to influence memory performance, by applying a targeted pulse of transcranial magnetic stimulation to the neuron network (see also Rose, 2020). This reactivation might constitute the neuronal correlate of the process known as refreshing (Barrouillet & Camos, 2015; Raye et al., 2007). This type of storage that disappears if not reactivated could explain why a concurrent processing demand pushed at its maximum leads to an almost complete loss of memory traces in delays of few seconds. Note that such a short-term forgetting does not preclude the creation of LTM traces, because the mechanisms of short-term plasticity might provide building blocks for potentiation mechanisms that support LTM through long-term modification of synaptic proteins and synaptic remodelling and growth (Dudai, 2004; Rose et al., 2016).

This conception is not so far from dual-store models inspired by Atkinson and Shiffrin’s (1968) modal model such as the search of associative memory model (SAM; Lehman & Malmberg, 2013; Raaijmakers & Shiffrin, 1980). These models assume a limited-capacity buffer storing and maintaining an episodic image of each of the studied items associated with the study context, some of these items being encoded into LTM. As in the modal model, episodic images in SAM model are maintained through control processes referred to as *rehearsal*. Because, in line with the modal model, information decays when not actively maintained, it is probable that all the items have disappeared from the buffer at the end of a trial in which a concurrent task has been performed at individual’s maximum capacity. Memory residual would thus be retrieved from LTM via contextual cues. However, delaying recall of 60 s would provoke a drift of the temporal context, decreasing its similarity with the study context, and consequently the probability of recall (see also Davelaar et al., 2005). This would explain the smaller memory residuals after the delay. Note that the small size of the memory residual in immediate recall suggests that the creation of LTM traces is rather sporadic, and does not exceed the single item estimated by Cowan et al. (2012).

To conclude, our study replicated Barrouillet et al.’ (2024) findings of a small memory residual under a quasicomplete attention distraction by a processing concurrent activity pushed at its individual’s maximum. The introduction of a delayed recall condition revealed reduced memory residuals after a 1-min concurrent activity. However, the size of these residuals after delay supports our hypothesis that memory residuals in WM mainly rely on LTM and provides empirical evidence for Cowan et al.’s (2012) estimation of an LTM contribution to one item in WM recall performance. Nonetheless, our findings seem difficult to reconcile with a greater implication of LTM through an offloading mechanism of memory traces under attentional capture, but seem more in line with the short-term transient storage hypothesized by the synaptic theory of WM (Mongillo et al, 2008; Rose, 2020).

## Data Availability

The preregistration and data sets generated during the current study are available via the Open Science Framework: https://osf.io/ad4bh/?view_only=84ee0b233480419d89038a6b5ca322eb

## Footnotes

1 This minimum *N* was determined from our previous studies on residuals, which indicated that this sample size is sufficient to reveal large effects (Barrouillet et al., 2024; Pougeon et al., 2024).

2 Order of the trials according to the pseudo-randomly assigned recall condition (immediate recall condition: ‘i’; delayed recall condition: ‘d’): ‘i’ (i.e., the first trial had an immediate recall), ‘i’, ‘d’, ‘i’, ‘d’, ‘d’, ‘d’, ‘i’, ‘d’, ‘d’, ‘i’, ‘i’, ‘d’, ‘d’, ‘d’, ‘i’, ‘i’, ‘i’, ‘d’, ‘i’, ‘d’, ‘i’, ‘i’, ‘i’, ‘d’, ‘d’, ‘i’, ‘d’, ‘d’, ‘d’, ‘i’, ‘i’, ‘d’, ‘i’, ‘i’, ‘d’, ‘i’, ‘i’, ‘d’, ‘d’ (i.e., the last trial had a delayed recall).

3 These data followed a normal distribution in the single task, *W* =.90, *p* =.05, and in the dual task for the immediate, *W* =.93, *p* =.13, and delayed recall condition, *W* =.95, *p* =.37. Skewness values were respectively 1.03, −.14, and −.26.

4 Shapiro–Wilk test indicated that the percentage of letters recalled in correct serial position did not follow a normal distribution, *W* = 0.85, *p* <.01, *W* = 0.79, *p* <.01 and *W* = 0.87, *p* = 0.01 in single task, dual task with immediate recall, and dual task with delayed recall, respectively. Additionally, the corresponding Skewness values were, respectively, −.89, 1.51, and 0.90.

5 These percentages of recall did not follow a normal distribution., *W* =.90, *p* =.05 (Skewness = −.57), and *W* =.88, *p* =.01 (Skewness = −1.08) for immediate and delayed recall condition.

6 Shapiro–Wilk tests indicated that the percentage of recall in the dual task for both immediate and delayed recall conditions deviated from a normal distribution, *W* =.82, *p* <.01, and *W* =.87, *p* <.02, respectively, but not in the single task, *W* =.93, *p* =.14. Additionally, the Skewness values were higher than 1, suggesting that these data in the dual task were asymmetrical (1.99 and 1.26 for immediate and delayed recall), but not in the single task (−0.71).

## Declarations

### Author note

This study was preregistered. Preregistration and data files are available via the Open Science Framework: https://osf.io/ad4bh/
